# Author's reply

**DOI:** 10.4103/0970-1591.36894

**Published:** 2007

**Authors:** A. Srivastava, M. S. Jha

**Affiliations:** Department of Urology and Renal Transplantation, Sanjay Gandhi Post Graduate Institute of Medical Sciences, Lucknow, UP, India. E-mail: anees772000@yahoo.com

Dear Sir,

We greatly appreciate your interest in the paper[1] and fully agree with your view that the small sample size in our study increased the probability of making a type I error.

We regret the typographical error in our paper whereby type I was mistyped as a type II error.
